# A Novel Approach to Predict Wrinkling of Aluminum Alloy During Warm/Hot Sheet Hydroforming Based on an Improved Yoshida Buckling Test

**DOI:** 10.3390/ma13051165

**Published:** 2020-03-05

**Authors:** Gaoshen Cai, Jubo Fu, Dongxing Zhang, Jinlin Yang, Yongfeng Yuan, Lihui Lang, Sergei Alexandrov

**Affiliations:** 1 Faculty of Mechanical Engineering & Automation, Zhejiang Sci-Tech University, Hangzhou 310018, China; caigaocan@zstu.edu.cn (G.C.); fujubo598@163.com (J.F.); jinlinyang@126.com (J.Y.); yuanyf@zstu.edu.cn (Y.Y.); 2 Department of Mechanical & Material Engineering, Western University, London, ON N6A5B9, Canada; 3 School of Mechanical Engineering and Automation, Beihang University, Beijing 100191, China; lang@buaa.edu.cn; 4 Institute for Problems in Mechanics of the Russian Academy of Sciences, 119526 Moscow, Russia; sergei_alexandrov@yahoo.com

**Keywords:** warm sheet hydroforming, hydromechanical deep drawing, wrinkling prediction, through-thickness normal stress

## Abstract

In order to predict the wrinkling of sheet metal under the influence of fluid pressure and temperature during warm/hot hydroforming, a numerical simulation model for sheet wrinkling prediction was established, taking into account through-thickness normal stress induced by fluid pressure. From simulations using linear and quadratic elements, respectively, it was found that the latter gave results that were much closer to experimental data. A novel experimental method based on an improved Yoshida Buckling Test (YBT) was proposed for testing the wrinkling properties of sheets under the through-thickness normal stress. A wrinkling coefficient suitable for predicting wrinkling was also presented. Based on the numerical simulations, an experimental validation of wrinkling performance was conducted. Ridge-height curves measured along the main diagonal tensile direction of the sheet were presented and showed that the wrinkling prediction criterion provided good discrimination. Furthermore, the wrinkling properties of several different materials were simulated to evaluate the accuracy of the prediction method, and the results revealed that the improved YBT gave good predictions for wrinkling in the conventional sheet metal forming process, while the prediction results for wrinkling in warm/hot sheet hydroforming were also accurate with the fluid pressure of zero.

## 1. Introduction

Warm/hot sheet hydroforming was proposed to enhance the plasticity of lightweight materials [1,2,3,4]. This innovative and flexible processing method combines the advantages of cold sheet hydroforming and warm/hot sheet forming [5,6,7] and is regarded as an advanced technique for improving material formability [8,9,10,11]. In contrast to the conventional sheet hot stamping process, fluid pressure provides the through-thickness normal stress and assists the sheet formation process during warm/hot sheet hydroforming [12,13]. Therefore, the stress states under the influence of fluid pressure are of great significance for elucidating the forming mechanism of this technique [14,15,16,17] and also play an important role in selection of processing method, parameter optimization, and determination of the rules governing deformation during forming [18,19]. Consequently, it is necessary to analyze how the fluid pressure acts on the sheet during the forming process [20,21].

Wrinkling instability is one of the main defects that arise during the sheet metal plastic forming process, and it can directly affect the forming quality and precision of thin-walled parts [22,23]. It can also increase wear on the forming die. Thus, the harmful effects of wrinkling on the forming of parts are obvious, and, in view of this, research on wrinkling prediction and control of sheet metal is a key topic in the field of plastic forming.

Although warm/hot sheet hydroforming can reduce the tendency to fracture and instability (wrinkling, cracking, etc.) [9,12], wrinkling can still occur during sheet forming. To improve the precision and quality of forming of parts and effectively eliminate wrinkling instability, it is particularly important to obtain accurate predictions of the likelihood of wrinkling in the forming process [24,25,26]. Therefore, it is necessary to investigate the prediction of wrinkling of sheet metal during warm/hot hydroforming.

## 2. Theory of Prediction of Sheet Wrinkling

### 2.1. Theoretical Prediction Model for Sheet Plastic Instability

To date, research on wrinkling has focused mainly on theoretical and experimental aspects. In terms of plastic instability theory, two basic mathematical models to predict wrinkling have been presented: The bifurcation model and the energy model [27,28,29]. The bifurcation model is based on a variational approach: When the Hill bifurcation functional has a nonzero solution, the material will wrinkle. The energy model is based on the principle of plastic work and compares the critical wrinkling energy with the plastic deformation work: When certain conditions are met, wrinkling will occur. These theoretical models can predict the wrinkling of typical parts during the forming process, such as the hydromechanical deep drawing of cylindrical cups and the hydraulic bulging of pipes under liquid pressure [30,31].

However, it is difficult to represent the surface shapes of complex thin-walled parts using simple real functions, and the boundary conditions in the forming process are very complicated. As a consequence, it is not possible to obtain analytical solutions of the equations resulting from the models in terms of real functions. In addition, the complicated nature of these equations means that errors may easily occur during calculations. 

Besides the preferred orientation and the relative thickness (*t/D*) of sheet metal, wrinkling is related to the stress ratio of deformation, the mechanical properties of the material, the geometric shape of the die, and the contact conditions between workpiece and die [25,26]. However, it is difficult to take account of all of these factors in a theoretical model. The development of finite element numerical simulation has provided an important approach for predicting wrinkling and developing measures for its control. In view of this, a prediction model for warm/hot sheet hydroforming based on the Yoshida buckling test (YBT) [32] was proposed in this study.

### 2.2. Prediction Method Based on a Sheet Plastic Instability Test

**Figure 1:**
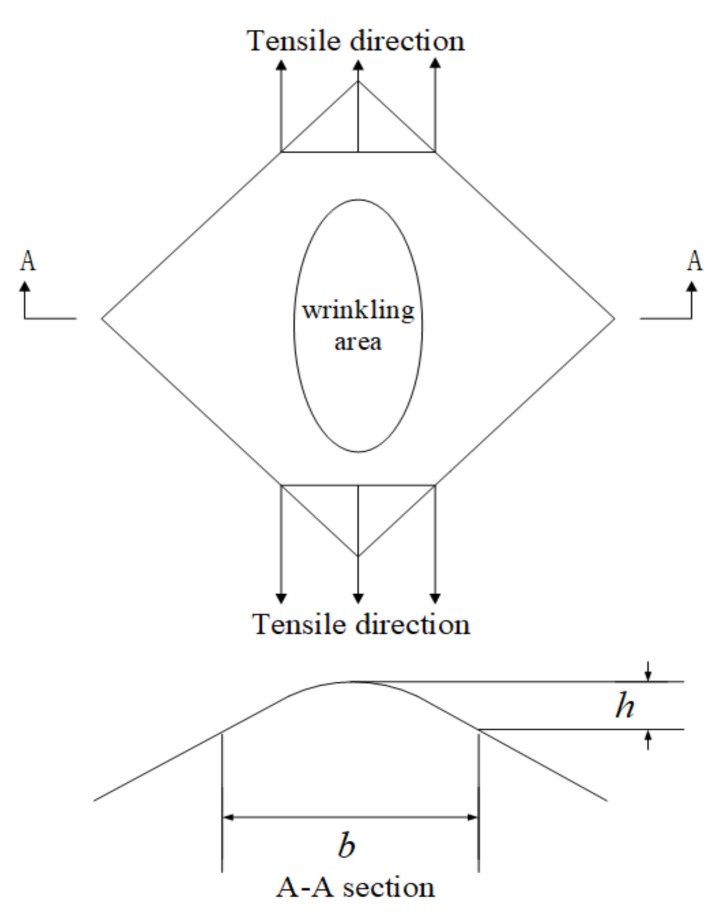
Schematic of the YBT.

Before theoretical methods had been established for the prediction of wrinkling in conventional sheet metal forming, experimental approaches, including the YBT proposed by Yoshida [32] in 1983 and the tube wrinkling property test proposed by Reddy, were generally used for qualitative analysis of the wrinkling properties of materials. The YBT, also known as the square sheet diagonal tensile test [33], is an experimental method for conducting a diagonal tensile test on a square sheet to evaluate the resistance to wrinkling due to compressional instability of a sheet materials in an uneven tensile state. Also, it has proved to be an effective simulation test of wrinkling properties [34], allowing qualitative analysis of the wrinkling tendencies of different materials. A schematic illustration of the test is shown in Figure 1, in which *b* is the center span of the basis of measurement (mm), and *h* is the height of wrinkling (mm).

It has been reported that [26,35] wrinkling inevitably accompanies compressive stress. In sheet plastic forming, when the compressive stress in one direction of a shell structure increases to a certain extent, if there is no supporting force in the through-thickness direction or if the supporting force is insufficient for the original equilibrium state to be recovered, then wrinkling will appear. In the deep drawing of a sheet, wrinkling is most likely to occur in the flange region of the blank. To reduce the possibility of wrinkling, the factors affecting wrinkling in the flange region should be considered when simulating the process of sheet metal forming.

### 2.3. Theoretical Basis of Control of Wrinkling

To prevent wrinkling during the forming of sheet metal, it is not sufficient just to predict its occurrence: It is also essential to analyze how it can be controlled during a practical machining process. It is necessary to start from the basic theory, study the relevant stability conditions, critical conditions, and basic stress-strain states, and then provide a theoretical basis for controlling wrinkling during sheet forming.

**Figure 2:**
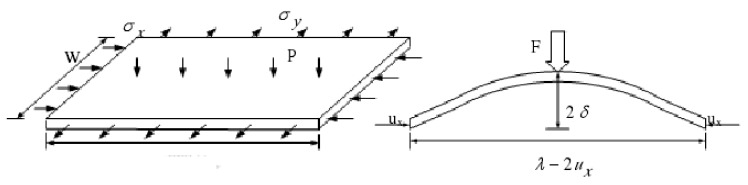
Schematic of DMV thin shell element.

A thin shell element according to Donnell-Mushtari-Vlasov (DMV) theory is shown in Figure 2, in which the 2*δ* is equivalent to *h* and $\lambda -2u_{x}$ is equivalent to *b* in Figure 1. In the plate buckling analysis, the strain deformation is small, based on the assumption that when the ratio of the wrinkling height to the thickness of the sheet is less than 0.2 (*δ/t* < 0.2), and the loading process before wrinkling is proportional loading, the waveform of the wrinkling is symmetrical. Then, from Timoshenko’s buckling theory, the stability condition of the sheet is as follows:

(1) ΔT≤ΔU

where *∆U* is the plastic buckling energy and *∆T* is the work done on the sheet by the external force. This condition indicates that the sheet is in a stable state when the work done by the external force does not exceed the plastic buckling energy, whereas if ΔT>ΔU, then wrinkling of the sheet will occur.

According to Hutchinson’s double-curvature shell element theory, the Lagrangian strain tensor at the neutral layer (*x*_3_) of the buckling instantaneous distance can be expressed as:

(2) $$\varepsilon_{\alpha \beta }=E_{\alpha \beta }+x_{3}\kappa_{\alpha \beta }$$

where α,β∈{1,2}. Here, $E_{\alpha \beta }$ and $\kappa_{\alpha \beta }$ are respectively the tensile and bending strains on the shell elements:

(3) $$E_{\alpha \beta }=\frac{1}{2}(u_{\alpha ,\beta }+u_{\beta ,\alpha })+b_{\alpha \beta }w$$

(4) $$\kappa_{\alpha \beta }=-w_{,\alpha \beta }$$

where *u_α_* are the components of the displacement in the *x*_1_ and *x*_2_ directions, *w* is the displacement normal to the surface of the sheet, *b_αβ_* is the curvature tensor of the neutral layer under wrinkling, and a subscript comma indicates a partial derivative (e.g., $u_{\alpha ,\beta }=\partial u_{\alpha }/\partial x_{\beta }$).

The constitutive equation involving all the variables is $\sigma_{\alpha \beta }={\bar{L}}_{\alpha \beta \kappa \gamma }\varepsilon_{\kappa \gamma }.$

The expressions for the resultant force and bending moment on the shell element are then

(5) $$N_{\alpha \beta }=\int_{-t/2}^{t/2}\sigma_{\alpha \beta }\;dx_{3}=t{\bar{L}}_{\alpha \beta \kappa \gamma }{\dot{E}}_{\kappa \gamma }$$

(6) $$M_{\alpha \beta }=\int_{-t/2}^{t/2}\sigma_{\alpha \beta }x_{3}\;dx_{3}=\frac{t^{3}}{12}{\bar{L}}_{\alpha \beta \kappa \gamma }K_{\kappa \gamma }$$

According to the assumption of a DMV shell element, the buckling strain capacity of sheet can be expressed as:

(7) $$\Delta U=\int_{S}\left(\int M_{\alpha \beta }d\kappa_{\alpha \beta }+\int N_{\alpha \beta }dE_{\alpha \beta }\right)dS$$

where *S* is the area where wrinkling occurs. Substituting of the expressions (5) and (6) for $N_{\alpha \beta }$ and $M_{\alpha \beta }$ into (7) then gives

(8) $$\Delta U=\frac{t}{24}\int_{S}{\bar{L}}_{\alpha \beta \kappa \gamma }w_{,\kappa \gamma }w_{,\alpha \beta }dS+\frac{t}{2}\int_{S}{\bar{L}}_{\alpha \beta \kappa \gamma }b_{\alpha \beta }b_{\kappa \gamma }w^{2}dS$$

Because the external force does work in the neutral layer,

(9) $$\Delta T=\frac{1}{2}\int_{S}(N_{11}w_{,1}^{2}+N_{22}w_{,2}^{2})dS$$

Wrinkling is considered to occur when ΔT=ΔU. This condition is the critical equation that is used to judge the wrinkling of a sheet, which was implemented in numerical simulation software to simulate and investigate the wrinkling of simple sheet parts to prevent wrinkling.

## 3. Wrinkling Prediction Model for Sheet Metal During Warm Sheet Hydroforming 

### 3.1. Wrinkling Test Method for Wrinkling of Sheet Metal During Warm Hydroforming

As mentioned above, the wrinkling resistance of a material is an important property affecting its behavior during forming. For conventional forming methods at normal temperature, the YBT has been used as the evaluation standard for the wrinkling property of materials [32]. However, there are great technical difficulties in testing the wrinkling resistance of materials in a forming environment where the stress normal to the sheet thickness is provided by a warm/hot fluid medium. In addition, appropriate test equipment and evaluation criteria are not available for conducting the corresponding tests. Besides, this method cannot be used to examine the wrinkling performance of sheets under through-thickness normal stress provided by fluid pressure, and it is not always appropriate for testing the wrinkling performance of materials at elevated temperature.

**Figure 3:**
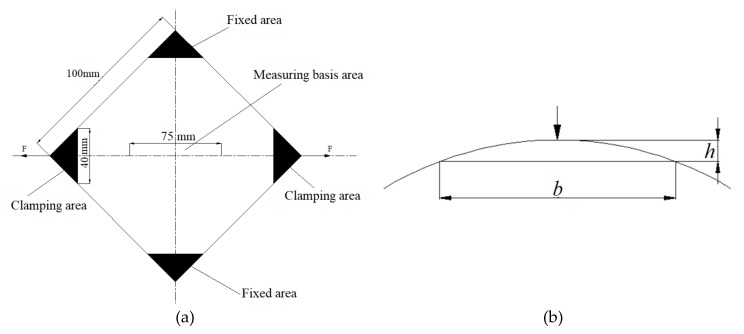
Schematic diagram of the wrinkling test. (**a**) Principle of the wrinkling test. (**b**) Basis of measurement.

To solve this technical problem and investigate the wrinkling performance of sheet metals during hydroforming, a novel approach to test the wrinkling performance of sheet metals under the through-thickness normal stress induced by fluid pressure has been proposed that is based on the YBT. This method can be conducted under high-temperature and high-pressure conditions, the square plate to be tested is placed in a special unit, and the through-thickness normal stress is applied to the sheet by means of fluid pressure. During the test, the elongation *λ*_75_ of a 75 mm region in the middle of the sheet is measured. When *λ*_75_ = 1%, the height *h* of the arch (i.e., the height of the wrinkling) is taken as the basic data. The corresponding center span is then about *b* = 25 mm. The curve of wrinkling height versus fluid pressure is then drawn from the recorded test data. A schematic illustration of this test method is shown in Figure 3.

**Figure 4:**
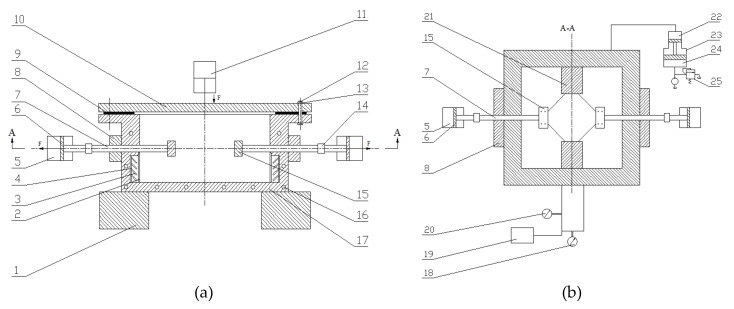
Structural diagram of sheet wrinkling test setup: (**a**) Front view; (**b**) cutaway view (A–A). 1, heel block; 2, swash partition; 3, heating unit; 4, insulating layer; 5, master cylinder; 6, master cylinder piston; 7, stretching rod; 8, support block; 9, high-temperature sealing ring; 10, case cover; 11, closed oil cylinder; 12, bolt; 13, nut; 14, displacement sensor; 15, sample chuck; 16, cooling hole; 17, tank body; 18, pressure measuring device; 19, data acquisition device; 20, temperature measuring device; 21, fixed mold; 22, high-pressure chamber; 23, fluid pressure generating device; 24, low-pressure chamber; 25, overflow valve.

In order to complete this test method, an appropriate experimental device was designed by the present authors. It consists of a closed box, a stretching mechanism, a fluid pressure system, a heating system, a cooling system, a pressure measuring system, a temperature measuring device, and a data acquisition and processing device, as shown in Figure 4.

The test method using this device involves the following main steps.

First, multiple groups of 100 mm ×100 mm square plate samples are prepared, and each of these is then divided into two fixed areas and two clamping areas (see Figure 3): The fixed areas are attached to the fixed mold inside the box, and the clamping areas are attached to the specimen holder, with a clamping width of 40 mm. A measuring reference area of length 75 mm is marked on the tensile axis of the sample. The relative elongation during stretching is denoted by *λ*_75_.

Second, the closed box is filled with oil by the fluid pressure system, and the heated oil is used to heat the samples to the set temperature, by which is to provide the high-temperature and the through-thickness normal stress conditions. Then two master cylinders are driven to stretch the specimen, and the stretching length is recorded by the displacement sensor. When *λ*_75_ reaches 1%, the test is stopped.

Third, the sample is cleaned briefly and data are recorded and processed. The arch height of the center span within *b* = 25 mm is denoted by *h*. From the processed data, curves of *h* versus the pressure in the oil chamber are drawn to provide a preliminary idea of the wrinkling behavior of the material.

Finally, the wrinkling coefficient $L_{\lambda }=(h/\lambda_{75})e^{p/p_{m}}$ is used as an index of wrinkling resistance: the greater the value of *L*_λ_, the less is the wrinkling resistance of the material, and the easier it is for the material to wrinkle. This coefficient directly indicates the degree of wrinkling of a material, and it provides a better reflection of the relationship between wrinkling and other factors than does the wrinkling height *H* obtained by the conventional YBT. This has important theoretical and practical ramifications for prediction of sheet metal wrinkling during warm/hot sheet hydroforming.

### 3.2. Wrinkling Prediction Method for Warm Sheet Hydroforming

In the test program, two indexes are used to measure the degree of wrinkling of sheet metal: One is the fluid pressure–wrinkling height curve, and the other is the previously mentioned wrinkling coefficient:

(10) $$L_{\lambda }=\frac{h}{\lambda_{75}}e^{p/p_{m}}\;\;(p,p_{m}\geq 0)$$

where *p* is the fluid pressure in the test procedure (MPa), *P*_m_ is the critical fluid pressure when the sheet is wrinkle-free (MPa), *h* is the measured wrinkling height (mm), and $\lambda_{75}$ is the relative elongation when the sheet is stretched in the reference zone, which is the tensile strain.

Equation (10) is the prediction model equation for sheet wrinkling adopted in this study. It shows that if no fluid pressure is applied during the test (*p* = 0), the wrinkling coefficient becomes $L_{\lambda }=h/\lambda_{75},$ which is the conventional YBT test method. In contrast, the approach presented here takes the fluid pressure and temperature into account, and is thus suitable for dealing with the test environment encountered in warm/hot sheet hydroforming. Therefore, the test data obtained using this method can reflect the wrinkling properties of sheets in the real forming process.

### 3.3. Numerical Simulation of Sheet Wrinkling Performance Test

By ignoring factors with only a slight influence, the wrinkling behavior of typical structural parts can be analyzed on the basis of an appropriate theoretical model, although for irregular parts with complex structures, it is difficult to establish such models. For practical application, it is necessary to discretize theoretical models with high precision, and in the present study, the finite element method was adopted to predict the occurrence of wrinkling.

#### 3.3.1. Numerical Calculation Method for Shell Elements

In finite element software, shell problems are generally divided into two types: Thick shell problems and thin shell problems. In addition, Abaqus software has available three different mathematical descriptions of general three-dimensional shell elements: General shell elements, thin-shell elements, and thick-shell elements (Table 1). These shell elements include linear and quadratic triangular elements and quadrilateral elements.

Isotropic material shells are considered to be thick shells when the ratio of thickness to span is greater than 1/15 and to be thin shells when this ratio is less than 1/15. In the case of the 100 mm × 100 mm square plate tensile test, the shell is thin. To verify the accuracy of wrinkling prediction using thin-shell elements, S4R and S8R, two element types were used to conduct numerical tests. 

#### 3.3.2. Influence of Different Microdisturbances on Simulation Results

A model for simulation was established, and the material selected was 2A16 aluminum alloy in the form of a square plate of side length *L* = 100 mm and thickness 1 mm. The clamping width was 30–50 mm, with a fixed constraint applied to the hydraulic chuck at the lower end of the test piece *B*, the upper portion and the rigid body *A* were connected by a tie constraint, the reference body control node RP-1 was assigned to the rigid body, and a displacement constraint along the tensile direction *Y* was applied to the control node.

According to the above analysis, since the thickness of the sheet used in the simulation was much less than the dimensions in the other two directions, the shell element in Abaqus was selected for the simulation. In finite element simulation, factors such as model size, flatness, surface finish, and chuck size can be all taken as ideal states, and shell elements could be affected only by the stress along the plane direction of the plate. It is therefore difficult to simulate deformation of the plate along the direction perpendicular to its thickness, and so wrinkling cannot be observed. Therefore, external factors can be considered as disturbances (concentrated forces and surface forces) applied to the plate.

(1) Concentrated Force Disturbance

**Figure 5:**
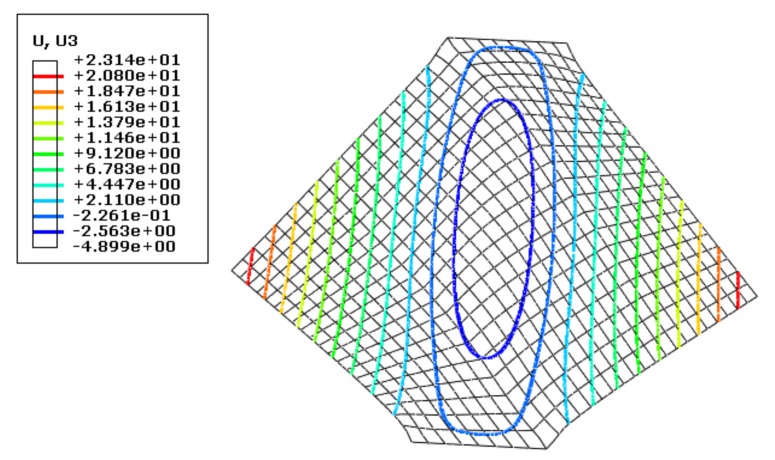
Simulation results for wrinkling of a sheet disturbed by a concentrated force.

In the simulation, a concentrated force (1 N) was applied very briefly to a corner of the non-clamping end of the sheet. The sheet size was again 100 mm × 100 mm, and the tensile displacement was 6 mm. Using a static algorithm, the simulation results of wrinkling height (mm) were obtained, as shown in Figure 5.

(2) Surface Force Disturbance

**Figure 6:**
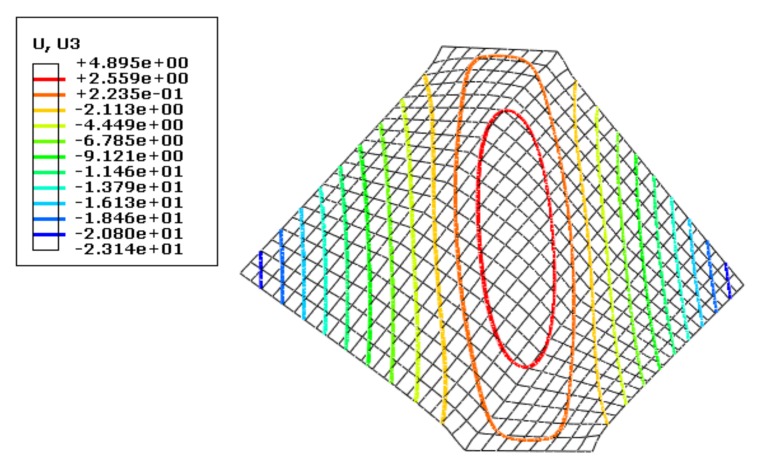
Simulation results for wrinkling of a sheet disturbed by a surface force.

By contrast, a very small (10^−3^ MPa) and uniformly distributed surface force was applied very briefly to the unclamped part of the sheet surface. The sheet size was also 100 mm ×100 mm, and the other settings were the same as before, then the simulation results were shown in Figure 6.

Figure 5 and Figure 6 indicate that when the sheet was disturbed by a concentrated force, the maximum wrinkling height at the center was 4.899 mm, while a disturbance by surface force under the same simulation conditions gave a value of 4.895 mm, a difference of just 0.004 mm, which shows the consistency of the simulation results under the two disturbance conditions. 

Both simulation and analytical results show that different forms of disturbance have similar effects on the wrinkling state of 2A16 aluminum alloy sheet.

#### 3.3.3. Influence of Different Algorithms on Simulation Results

(1) Static Algorithm Analysis

(i) Selection of Element Integral Mode

Abaqus software provides linear and second-order elements for the different shell element types. The linear element consumes less computational resources and has better computational stability in the analysis of complex friction contact processes. However, in the sheet buckling problem, the shell element reference surface will be subject to continuous curvature deformation. To accurately describe the consequent changes in shape, it is necessary to make appropriate choices of mesh division and element type. Situation results show that quadratic elements provide a better expression of the deformation of the surface boundary. Besides, the calculation of the grid with the linear element was faster than with the quadratic element. The reason for this is the greater number of integral nodes in the quadratic element, which makes the arithmetic and integration processes more complicated.

(ii) Choice of Meshing Method

The meshing density and partitioning method are related to the accuracy of the simulation results. Therefore, an appropriate choice of mesh density is an important aspect of the simulation. To ensure consistency with the simulation settings described above, the clamping width of the sheet was taken as 30 mm and the stretching stroke was taken as 5 mm in the simulations. Using the S8R element, simulation results for the wrinkle height with different grid sizes are shown in Table 2.

The simulation results show that the calculation speed became faster with the larger grid size divided on the sheet. Conversely, the smaller grid size resulted in the slower calculation speed. In addition, the simulation results with grid sizes of 2 mm and 4 mm were similar, although for the quadratic element, the finer meshing gave rise to the smaller overall stiffness of the shell and the greater wrinkle height.

(2) Dynamic Algorithm Analysis

Prediction of wrinkling with a dynamic algorithm commonly used for the forming process of sheet was studied. The sample size was again 100 mm × 100 mm, the clamping width was 30 mm, the tensile displacement was 5 mm, and the loading time was 1 s. To determine whether the simulation process was quasi-static, it was necessary to check whether the kinetic energy of the analyzed body was less than the internal energy by several percentage points in the post-processing result.

**Figure 7:**
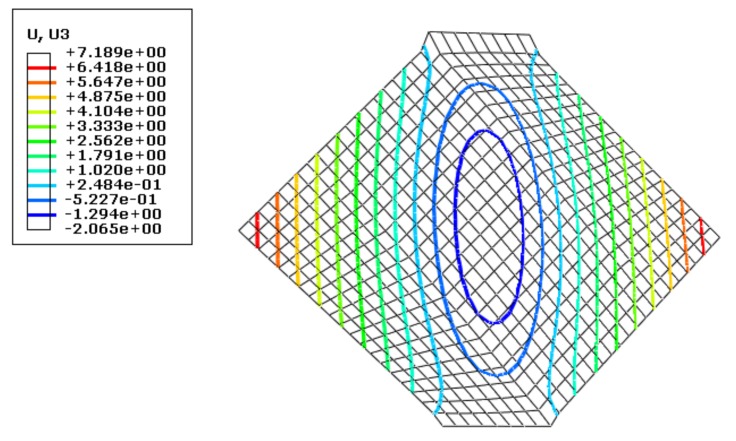
Deformation nephogram using the dynamic algorithm with a grid size of 4 mm.

**Figure 8:**
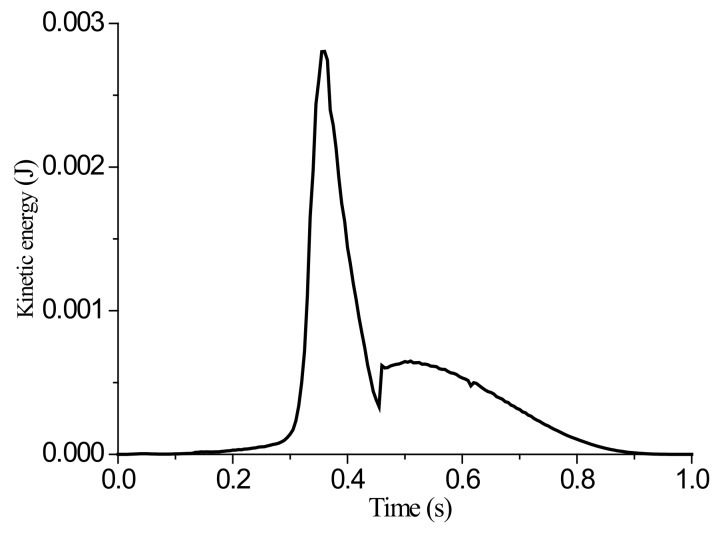
Variation of kinetic energy with time.

**Figure 9:**
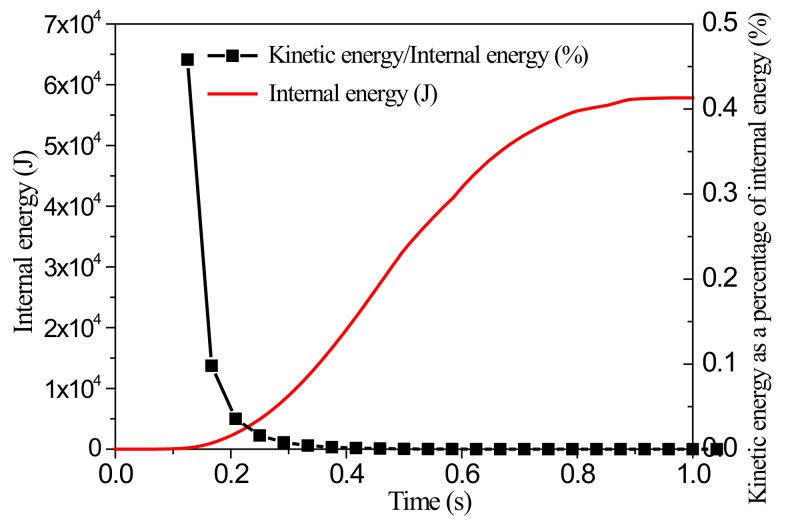
Variations of internal energy and of ratio of kinetic energy to internal energy with time.

As mentioned above, the simulation results for grid sizes of 2 mm and 4 mm were in accordance. Therefore, subsequent simulations were performed for both these sizes. Figure 7 shows the results of the wrinkling height (mm) for a grid size of 4 mm, Figure 8 shows the corresponding variation of kinetic energy with time, and Figure 9 shows the variations of internal energy and kinetic/internal energy ratio with time.

Simulation results indicate that the proportion of kinetic energy in the internal energy remained below 0.5% throughout the loading, and so the whole simulation can be considered to be a quasi-static loading process. When the final stretching stroke was 5 mm, the wrinkling height at the center of the sheet was 2.065 mm.

**Figure 10:**
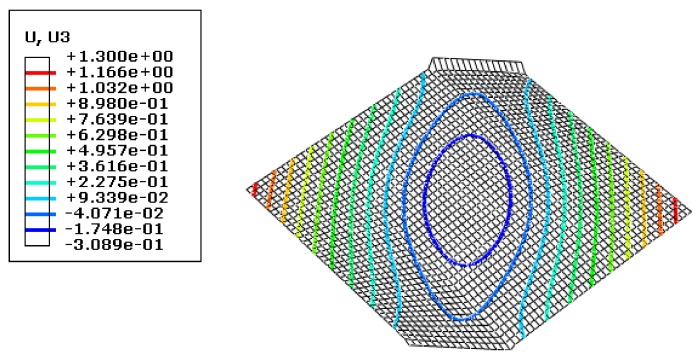
Deformation nephogram using the dynamic algorithm with a grid size of 2 mm.

**Figure 11:**
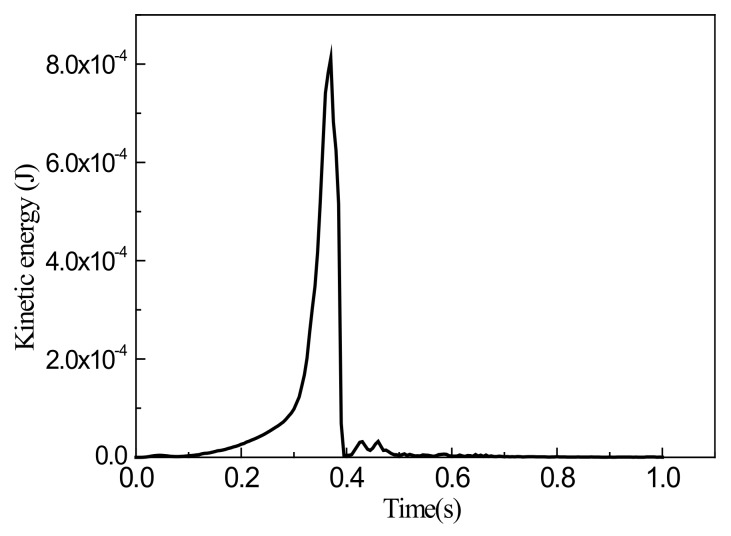
Variation of kinetic energy with time.

**Figure 12:**
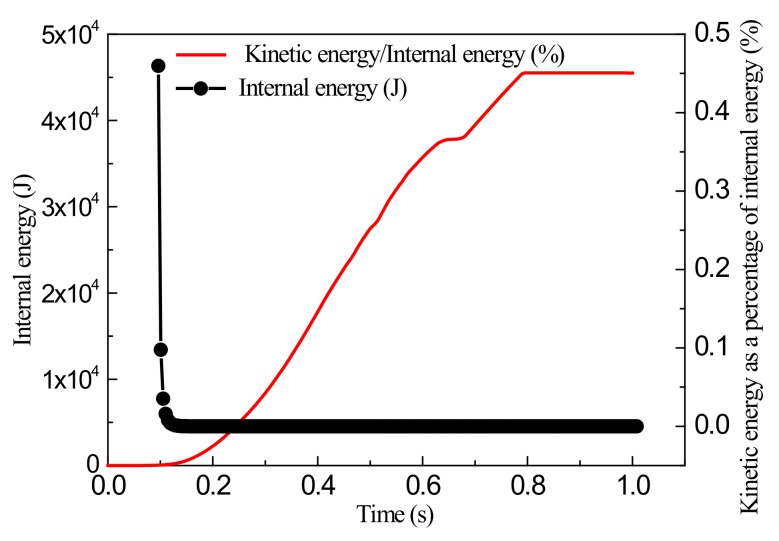
Variations of internal energy and ratio of kinetic energy to internal energy with time.

For comparison, the simulation was also performed for a grid size of 2 mm, and the simulation results were obtained, as shown in Figure 10, Figure 11 and Figure 12. 

Results show that for a grid size of 2 mm, the proportion of kinetic energy was still below 0.5% during the entire loading process, and this process can again be considered to be quasi-static. Also, when the stretching stroke was 5 mm, the wrinkling height at the center of the sheet was 0.309 mm, which is significantly smaller than measured by the test. A comparison with the simulation results with a grid size of 4 mm shows that the simulation results are in accordance. This verifies the above analytical results regarding the selection of grid size. Furthermore, the simulation results are in accordance with those of the theoretical analysis, which proves the reliability and applicability of the simulations.

### 3.4. Experimental Verification of Sheet Wrinkling Properties

To verify the accuracy of the above theoretical and numerical simulation results, an experiment was carried out.

#### 3.4.1. Test Equipment and Material

**Figure 13:**
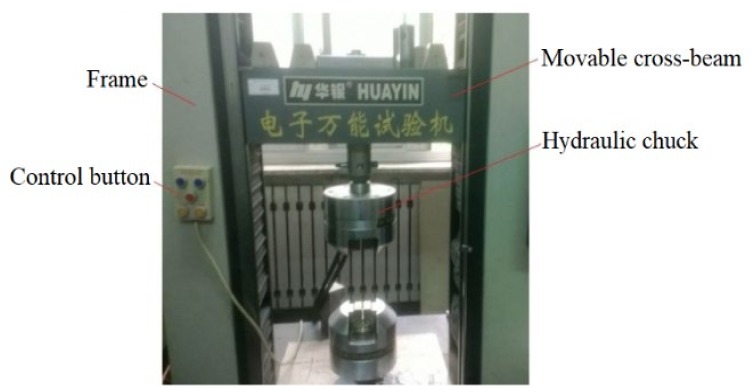
Electronic universal testing machine.

In its unfilled state (without fluid pressure), the equipment used for the sheet wrinkling test includes an electronic universal testing machine, chucks (a pair with widths of 50 mm and 80 mm, respectively), and an extensometer, as shown in Figure 13 (WDW-100, Wuxi Huayin Test Instrument CO. LTD, Wuxi, China). At the center of the equipment is the operating part, used for assembling the chuck and samples. The clamping is hydraulic, and the operation is controlled by computer, using TE engineering software. During the test, this software was used to control the rise and fall of the moving beam and input parameters such as the test tensile speed, as well as dynamically displaying the test process and the stress–strain curves.

**Figure 14:**
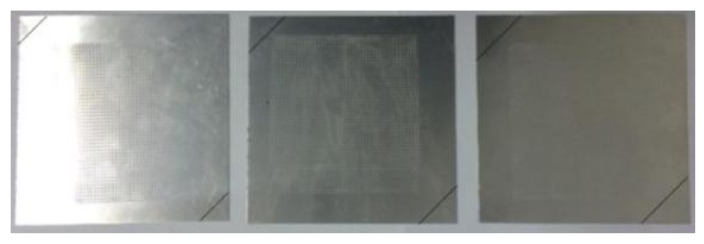
Samples before wrinkling.

The material used in this test was 2A16 aluminum alloy sheet. The samples were 100 mm × 100 mm square plates of thickness 1.0 mm. As shown in Figure 14, the diagonal lines drawn on the corners of the plates indicate the places where they were clamped by the chucks. The clamping widths were 30 mm, 40 mm, and 50 mm.

#### 3.4.2. Analysis of Experimental Results

**Figure 15:**
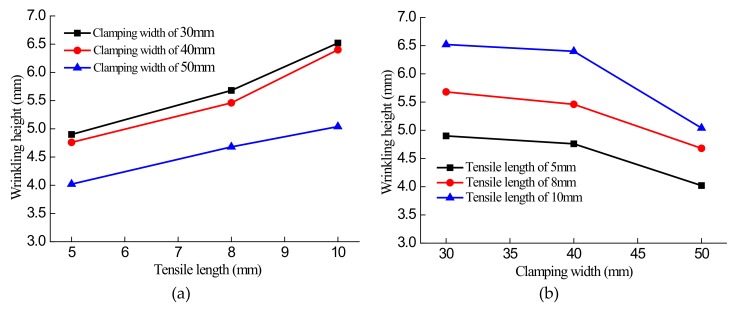
Test curves for wrinkling height: (**a**) Wrinkling height versus tensile length for different clamping widths; (**b**) wrinkling height versus clamping width for different tensile lengths.

The wrinkling test was carried out at room temperature, and the wrinkling height was measured as the relative height at the center of the equidistant width *L* = 25 mm at the intermediate section, using a height measuring instrument. To further verify the trend of variation of the wrinkling height, the measurements obtained were plotted in Figure 15 versus clamping width and tensile length to show the dependences of the wrinkling height on these parameters.

Figure 15a shows that for a given value of the clamping width, the wrinkling height of the samples increased as the tensile length increased from 5 mm to 8 mm to 10 mm. Figure 15b indicates that for a given value of the tensile length, the wrinkling height decreased as the clamping width of the samples increased from 30 mm to 40 mm to 50 mm, which means that the wrinkling becomes less and less obvious.

Experimental results indicate that, in general, the wrinkling tendency of warm/hot sheet hydroforming is that the wrinkling height of the sheet decreases with increasing sample clamping width, and it increases with increasing tensile length. Also, the results verify the correction of the numerical simulation of sheet wrinkling performance.

#### 3.4.3. Surface Detection in the Main Diagonal Direction of Test Samples

**Figure 16:**
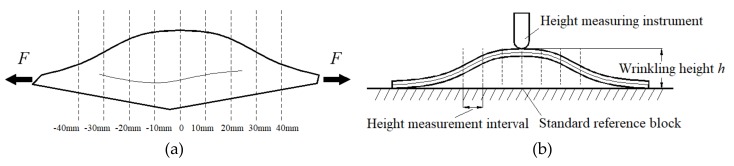
Wrinkling height measurement for surface detection: (**a**) Wrinkling height in the diagonal direction; (**b**) schematic of measurement procedure.

There are insufficient measured data on wrinkling height according to the national standard to fully characterize the wrinkling of different samples and determine the wrinkling susceptibility of different materials. Therefore, to evaluate the accuracy of the numerical simulation in predicting the wrinkling failure mode of a material, the wrinkling height *h* was measured at intervals of 10 mm from the center of a square sheet sample in the diagonal direction and was then plotted versus the diagonal ridge line, as shown in Figure 16.

In these measurements, the imaginary mid-surface of the shell was taken as the measurement surface. According to small-deformation theory, the assumption that the deformation of the sheet was uniform and variations in the thickness of the sheet before and after tension were negligible was applied. The wrinkling height at each measurement point is then:

(11) H=h−t

where *H* is wrinkling height of the mid-surface (mm), *h* is the measured wrinkling height (mm), and *t* is the sheet thickness (mm). In this test, *t* was 1 mm.

**Figure 17:**
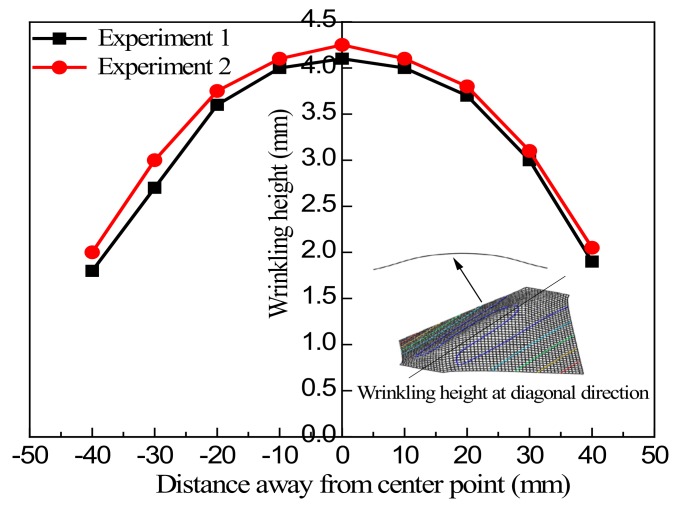
Comparison of measurements of wrinkling height of samples in the main diagonal direction.

In this test, two group samples with side length *L* = 100 mm, clamping width *B* = 30 mm were selected for measurement, with a tensile stroke *d* = 5 mm. The wrinkling height curves of the two group samples along the tensile diagonal direction under the same loading conditions were obtained, as shown in Figure 17.

Figure 17 shows that the results for the two samples were not completely consistent, and there was a deviation of about 2%. There are probably a number of reasons for this, the most likely being that it was due to errors in the test and measurement procedures. To improve the accuracy of experimental data to allow these to serve as a basis for sample comparison, the average value of two tests can be taken, and this was done to verify the subsequent numerical simulation results.

#### 3.4.4. Comparison of Calculation Results

**Figure 18:**
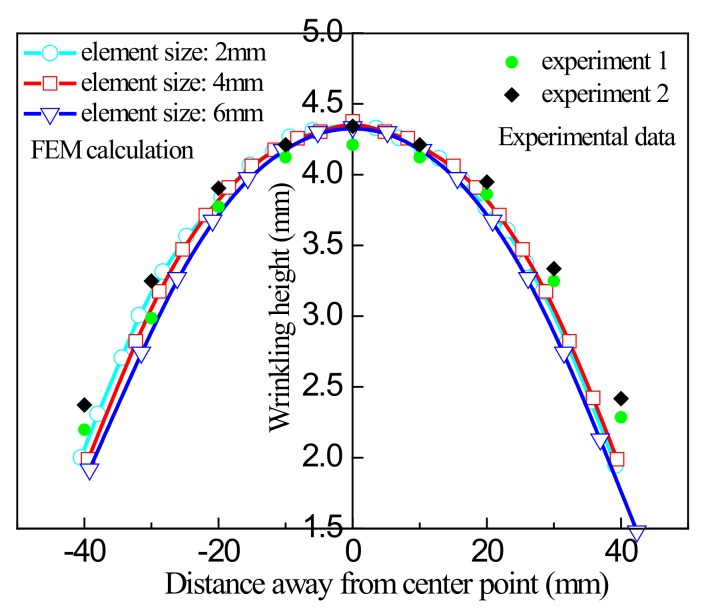
Comparison of the results of simulation using the S8R quadratic element and experimental data.

**Figure 19:**
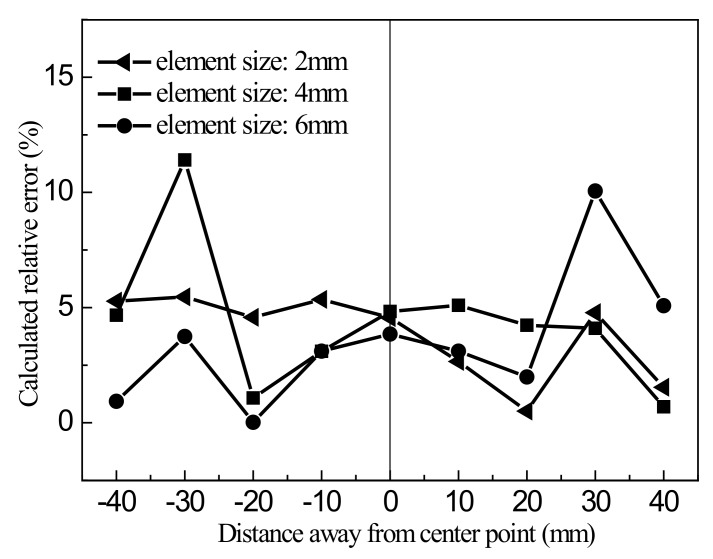
Relative error in the simulation results using the S8R quadratic element.

To evaluate the accuracy of different element algorithms in predicting the wrinkling form of sheets, the shape distribution of ridge lines in the main diagonal direction was studied for various simulation conditions, using the surface detection method along the main diagonal described above. Figure 18 shows the results of simulation using the static implicit algorithm with the S8R quadratic element compared with experimental data, and Figure 19 shows the relative error in the results from this algorithm.

Figure 18 and Figure 19 indicate that for the static simulation algorithm with quadratic elements, the size of the elements has an impact on the simulation results. For an element size of 2 mm, the relative error in the simulation results is about 5%. When the element size is increased to 6 mm, the maximum relative error exceeds 10%. In general, comparison of experimental data and simulation results shows that the static algorithm using quadratic elements has higher accuracy in numerical simulation than the other algorithms, and its results are in good agreement with experimental data.

**Figure 20:**
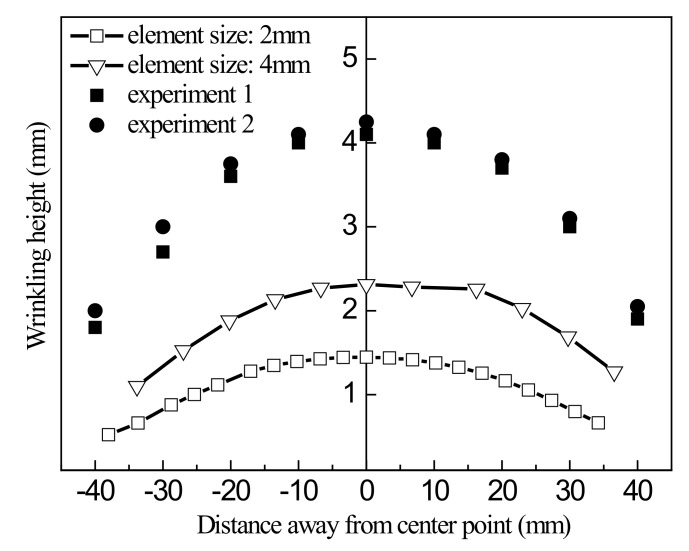
Comparison of simulation results using the S4R linear element and the static algorithm with experimental data.

**Figure 21:**
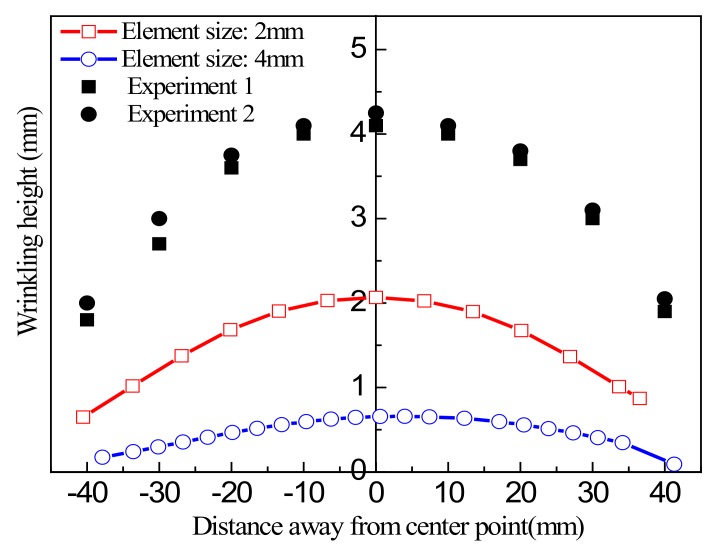
Comparison of simulation results using the S4R linear element and the dynamic algorithm with experimental data.

To compare the accuracy of simulation of sheet wrinkling prediction with different algorithms, a simulation using the S4R linear element with the static algorithm was also conducted. The simulation results are compared with experimental data in Figure 20, and Figure 21 shows the results of simulation using the dynamic algorithm with the S4R linear element compared with experimental data.

Figure 20 shows that the calculation accuracy with the linear element is very poor. For an element size of 2 mm, the error in the highest point is 65.7%. Therefore, it is difficult for an algorithm using the S4R linear element to accurately predict wrinkling during sheet forming process. Figure 21 indicates that the error is even larger than with the static algorithm and that it gets worse with decreasing element size. This further confirms that the S4R element is not suitable for use in element predicting wrinkling in the sheet forming process.

### 3.5. Evaluation of Accuracy of Sheet Wrinkling Test

On account of the YBT has long been the basic test to measure the wrinkling properties of sheets, to verify the accuracy of the modified YBT, tests on different materials were conducted. Sheets of different materials have different wrinkling heights under the same conditions, and so numerical simulations of wrinkling properties and comparisons of wrinkling heights on the ridge line in the main tensile direction of different materials were carried out.

**Figure 22:**
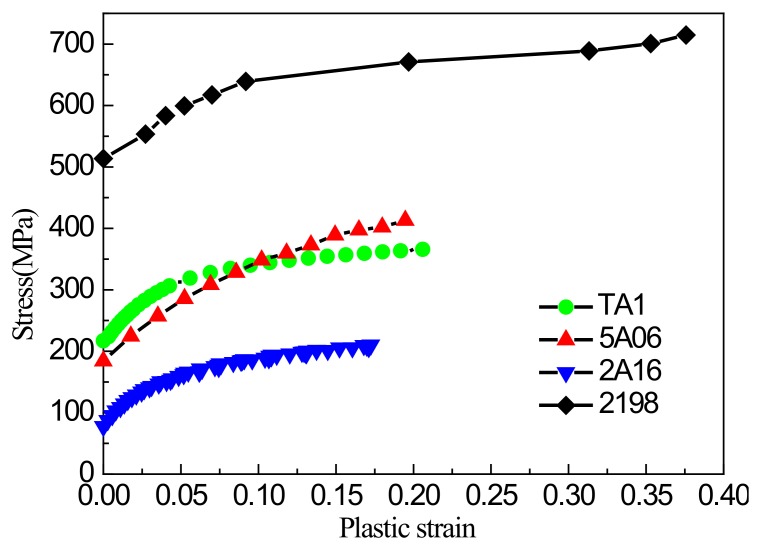
Stress-strain curves of several materials.

Wrinkling tests on sheets of 5A06 and 2A16 aluminum alloys, 2198 aluminum-lithium alloy, and TA1 titanium alloy were performed to evaluate the wrinkling performance of these materials. Stress–strain curves at normal temperature are shown in Figure 22.

**Figure 23:**
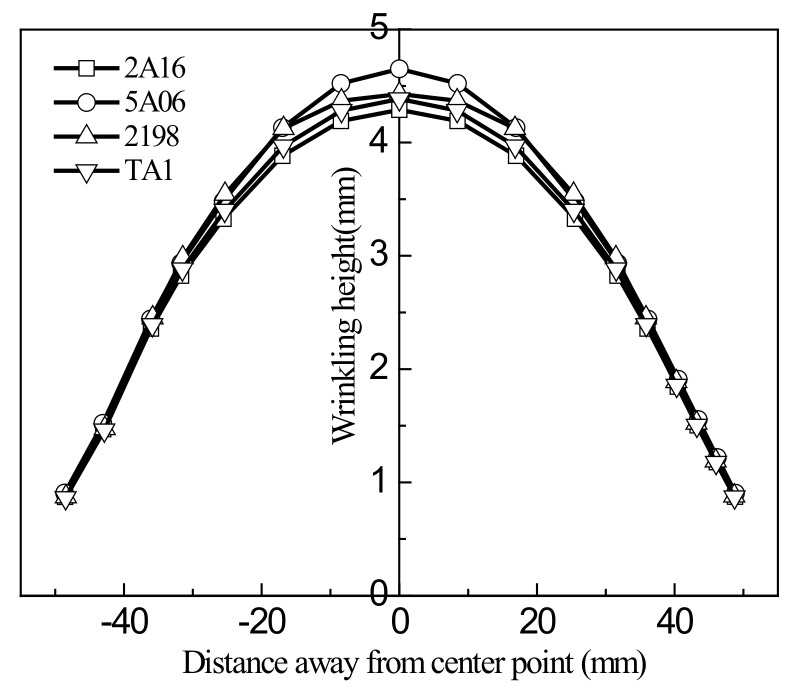
Ridge line height versus center distance in the principal tensile direction.

**Figure 24:**
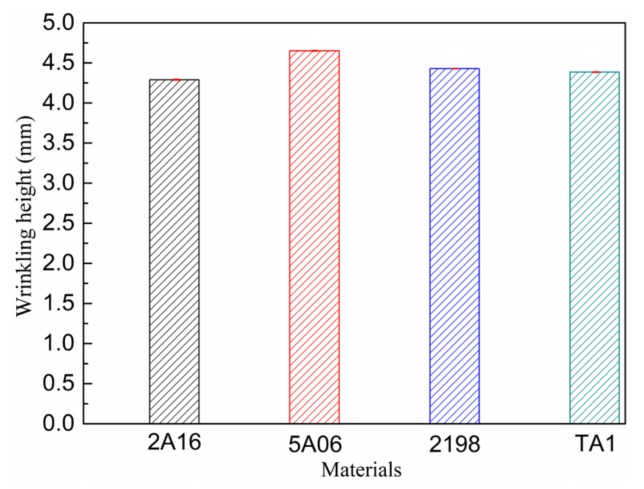
Maximum wrinkling height distributions of different materials.

The S8R shell element algorithm was again used for the simulations. The samples were 100 mm × 100 mm square sheets of thickness 1.0 mm, and the clamping width was 30 mm. The tensile stroke was 5mm, and the other test conditions were as in the earlier experiments. Curves of ridge line height versus center distance in the main tensile direction are shown in Figure 23, and the maximum wrinkling height distributions are shown in Figure 24.

Figure 23 and Figure 24 show that the behavior of the wrinkling height was similar for the different materials, although there were some differences. The 5A06 aluminum alloy sheet had the poorest resistance to wrinkling, and its maximum wrinkling height was the greatest. The 2198 aluminum-lithium alloy sheet had the greatest resistance to wrinkling, and its wrinkling height distribution was the most ideal. The difference in maximum wrinkling height among the materials was about 4.39%, so it can be considered that YBT performs well in predicting and evaluating the wrinkling of sheets.

These results show that the YBT is of great importance for predicting wrinkling of sheets. They also show that the proposed equation for predicting wrinkling of sheets is suitable for warm/hot sheet hydroforming when the fluid pressure is zero. This can provide a basis for subsequent study of the prediction of sheet wrinkling under fluid pressure and temperature, as well as providing basic test data for the further study of wrinkling during the forming process.

## 4. Conclusions

Using a novel approach based on an improved YBT, wrinkling of sheets in warm/hot sheet hydroforming was predicted using theoretical analysis, numerical simulation, and experimental verification. The general conclusions of this study can be summarized as follows:

(1) A novel experimental method based on an improved YBT was proposed to test the wrinkling properties of sheets under through-thickness normal stress provided by fluid pressure at different temperatures. A wrinkling coefficient *L_λ_* that expresses the ability of a sheet to resist wrinkling during the hydroforming process was also presented.
(2) The wrinkling test using the proposed method for sheet wrinkling properties was carried out, and results indicated that the wrinkling height of the sheet decreased with the increasing sample clamping width, and it increased with the increasing tensile length.
(3) Based on numerical simulation, the measurement method that Ridge-height curves measured along the main diagonal tensile direction of sheets was presented. Results indicated that the wrinkling prediction criterion allows good discrimination.
(4) The wrinkling properties of several different materials were simulated and analyzed, and the results showed that the improved YBT gave good predictions for wrinkling in the conventional sheet metal forming process, while the prediction results for wrinkling in warm/hot sheet hydroforming were also accurate when the fluid pressure was zero.

## Figures and Tables

**Table 1:** Abaqus shell element types.

General Shell Elements	Thin-Shell Elements	Thick-Shell Elements
S4R, S3R, SAX1, SAX2, SAX2T	STRI3, STRI35, STRI65, S4R5, S8R5, S9R5, SAXA	S8R, S8RT

**Table 2:** Comparison of calculation results for different grid sizes with the S8R element.

Grid Size (mm)	2	4	6
Wrinkle height at the center of the sheet (mm)	4.387	4.377	4.336
